# Prevalence of Thyroid Disease in Patients Surgically Treated for Pituitary Disease

**DOI:** 10.3390/jcm8081142

**Published:** 2019-07-31

**Authors:** Daham Kim, Yongin Cho, Cheol Ryong Ku, Hyein Jung, Ju Hyung Moon, Eui Hyun Kim, Dong Yeob Shin, Sun Ho Kim, Eun Jig Lee

**Affiliations:** 1 Department of Internal Medicine, Yonsei University College of Medicine, Seoul 03722, Korea; 2 Institute of Endocrine Research, Yonsei University College of Medicine, Seoul 03722, Korea; 3 Department of Endocrinology and Metabolism, Inha University School of Medicine, Incheon 22212, Korea; 4 Department of Neurosurgery, Yonsei University College of Medicine, Seoul 03722, Korea; 5 Department of Neurosurgery, Ewha Womans University College of Medicine, Seoul 03760, Korea

**Keywords:** prevalence, thyroid disease, hypothyroidism, hyperthyroidism, thyroid cancer, pituitary disease

## Abstract

Thyroid disease mainly has a thyroid origin but can occasionally have a pituitary origin. Clinicians face several challenges when these conditions occur together. We aimed to determine the prevalence of thyroid disorders in patients undergoing trans-sphenoidal adenomectomy (TSA) for pituitary disease. We reviewed the medical records of patients undergoing TSA for pituitary disease between 2008 and 2017 at Severance Hospital. Thyroid disorders were categorized using blood test results and medical histories at the time of preoperative evaluation. Among 2202 patients, 44 (2%), 218 (9.9%), and 74 (3.4%) had hyperthyroidism, hypothyroidism, and post-thyroidectomy status before TSA, respectively. Among the 44 patients with hyperthyroidism, 30 (68.2%) had central hyperthyroidism. Among the 218 patients with hypothyroidism, 165 (75.7%) had central hypothyroidism. Central hypothyroidism was more common in patients with adrenocorticotropic hormone-secreting pituitary adenomas (aOR (adjusted odds ratio) 1.85), Rathke’s cleft cysts (aOR 2.34), and craniopharyngiomas (aOR 2.58) (all *p* < 0.05) than in those with nonfunctioning pituitary adenomas. Contrastingly, thyroid cancer had an increased prevalence in patients with growth hormone- (aOR 3.17), prolactin- (aOR 3.66), and thyroid-stimulating hormone-secreting (aOR 6.28) pituitary adenomas (all *p* < 0.05). Pituitary disease sometimes accompanies thyroid disorders; their characteristics vary according to the type of pituitary disease.

## 1. Introduction

The thyroid gland produces thyroid hormones that play a critical role in maintaining thermogenic and metabolic homeostasis. Thyroid hormone output is regulated by the thyroid-stimulating hormone (TSH) secreted by the anterior pituitary gland; TSH is regulated by the thyrotropin-releasing hormone (TRH) produced by the hypothalamus [1]. Therefore, thyroid disorders can be caused by a defect anywhere in the hypothalamic-pituitary-thyroid axis. 

Thyroid function tests, used to evaluate the cause of thyroid disease [2], are common in clinical settings. In most cases, interpreting thyroid function tests that evaluate free thyroxine (fT4), triiodothyronine (T3), and TSH is straightforward. However, there are still important pitfalls and difficult cases. Sometimes it is difficult to distinguish between a pituitary-origin and thyroid-origin thyroid disorder, especially when pituitary and thyroid diseases occur together. Although central hyperthyroidism and hypothyroidism are rare diseases, this difficulty can raise several challenges for clinicians [3,4]. In addition, in cases involving pituitary disease, physicians tend to focus on the pituitary problem, neglecting the thyroid disease, even though the treatments and prognoses for both conditions are different. 

Recently, there have been reports of thyroid cancer coexisting with TSH-secreting [5] or growth hormone (GH)-secreting pituitary adenomas [6]. However, although pituitary disease may affect thyroid cancer development, the rarity of the disease limits further study.

We aimed to investigate the prevalence of thyroid disease in patients who underwent trans-sphenoidal adenomectomy (TSA) for pituitary disease.

## 2. Experimental Section

### 2.1. Patients

We reviewed the medical records of patients who underwent TSA for pituitary disease from 2008 to 2017 at Severance Hospital, of whom 2202 patients were enrolled. Data on age, sex, and current medications were recorded. Medical histories were taken for all patients scheduled for TSA in our institute. At the same time, medical records from other institutes and information on current medications were also collected. History of thyroid disease was documented from the obtained hospital records. All thyroid disorders, including thyroid abnormalities (based on blood test results or medical histories at preoperative evaluation), were confirmed by an endocrinologist before TSA.

TSA for each classification of pituitary adenomas was performed according to clinical guidelines [7,8,9,10,11]. In brief, all patients with GH-secreting pituitary adenomas, Cushing’s disease, and TSH-secreting pituitary adenomas received TSA, except for cases of contraindication of general anesthesia. For prolactinoma, TSA was performed when dopamine agonists were ineffective in normalizing prolactin levels or when they led to intolerable side effects. Furthermore, patients with prolactinoma who preferred surgical resection to medical treatment or who desired to become pregnant soon received TSA. Lastly, clinically nonfunctioning pituitary adenomas and Rathke’s cleft cysts were surgically removed in cases where visual field defects were caused, thus approaching towards optic chiasm or enlarging progressively. This study was conducted in accordance with the 1964 Declaration of Helsinki and was approved by our institutional review board (No. 4-2018-0582); the need for informed consent was waived because of the retrospective nature of the study.

### 2.2. Definitions of the Diseases

Serum TSH (normal range, 0.35-4.94 μIU/mL), fT4 (normal range, 0.70–1.48 ng/dL), and T3 (normal range, 0.58–1.59 ng/mL) levels were measured using a microparticle chemiluminescence immunoassay (Abbott Ireland Diagnostics Division, Longford, Ireland). TSH receptor (TSHR) antibody (normal range, 0–1.75 IU/L) levels were measured using a third-generation TBII electrochemiluminescence immunoassay (Elecsys/Cobas; Roche Diagnostics, Mannheim, Germany) [12] in patients with hyperthyroidism.

Thyroid diseases were categorized into five groups: primary hyperthyroidism, central hyperthyroidism, primary hypothyroidism, central hypothyroidism, and post-thyroidectomy status. Primary hyperthyroidism was defined as a low TSH level with high fT4 (or T3) levels or current use of medication, including propylthiouracil, methimazole, or carbimazole, after confirmed TSHR antibody elevation. Based on the presence or absence of elevated TSHR antibody levels, primary hyperthyroidism was reclassified as Graves’ disease or transient thyrotoxicosis. Transient thyrotoxicosis was defined as a low TSH level with high fT4 (or T3) levels; (1) without evidence of thyroid autoimmunity, (2) a lack of Graves’ disease physical findings, and (3) spontaneous resolution. Central hyperthyroidism was defined as a normal or high TSH level with high fT4 (or T3) levels. Primary hypothyroidism was defined as a high TSH level with low fT4 (or T3) levels or a use of thyroid hormones (levothyroxine, liothyronine, or a combination of levothyroxine and liothyronine) for more than 6 months, in order to exclude patients with transient or central hypothyroidism. Central hypothyroidism was defined as low fT4 (or T3) levels without TSH elevation. Post-thyroidectomy status was separately defined as a medical history of thyroidectomy due to a benign or cancerous lesion. If medical history or thyroid function test results were ambiguous, such as in cases of iatrogenic thyrotoxicosis, the disease category was decided by two endocrinologists (Daham Kim and Yongin Cho). Cases of subclinical thyroid dysfunction, such as subclinical hyperthyroidism and subclinical hypothyroidism, were not defined as a disease category because origins of such cases were often unclear with respect to the presence of pituitary disease. In these cases, we followed up with regular thyroid function tests according to guidelines and considered further workup or treatment as necessary. Patients with GH adenomas who received preoperative treatment of somatostatin analogues underwent TSA after 3 months of last somatostatin analogues injection.

The diagnosis of pituitary disease was based on clinical and pathological findings. Most pituitary diseases were pituitary adenomas; other disorders, such as Rathke’s cleft cysts and craniopharyngiomas, were rare. Patients with GH adenomas further underwent thyroid ultrasonography due to the possibility of an increased incidence of thyroid cancer in patients of acromegaly.

### 2.3. Statistical Analysis

SPSS Statistics Version 23 (IBM Corp., Armonk, NY, USA) was used for all statistical analyses. The analyses of current study are retrospective and exploratory, and we did not calculate the sample size for our analyses. Categorical variables were presented as numbers and percentages, and continuous variables were presented as means ± standard deviations. The two-sample Student’s *t*-test was used to compare mean values; group comparisons were performed using the χ2 test, Fisher’s exact test, or linear-by-linear association, as appropriate. Multiple logistic regression analysis was performed to evaluate the differences in thyroid disease prevalence according to the type of pituitary disease. Age and sex were calibrated in adjusted models. Adjusted odds ratios (aORs) and 95% confidence intervals (CIs) were determined. *p*-values of <0.05 were considered statistically significant.

## 3. Results

### 3.1. Prevalence of Thyroid Disease Before TSA

**Figure 1:**
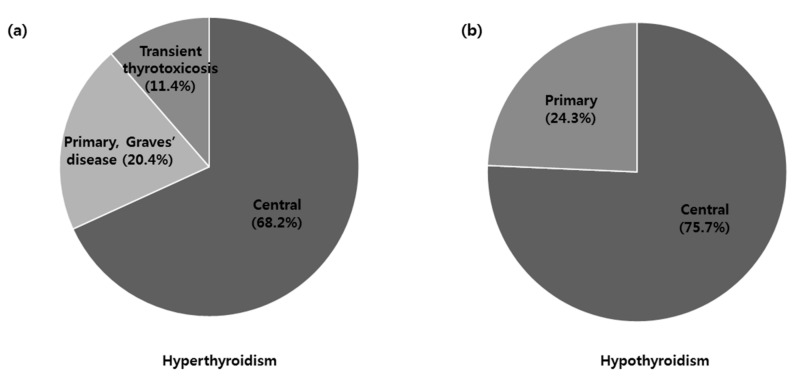
Classification of study subjects according to the presence of hyperthyroidism or hypothyroidism. (**a**) Among 44 patients with hyperthyroidism, 9 (20.4%) were confirmed to have Graves’ disease, and 5 (11.4%) were confirmed to have transient thyrotoxicosis. (**b**) Among 218 patients with hypothyroidism, 53 (24.3%) were confirmed to have primary hypothyroidism. The remaining patients had central hypothyroidism.

A total of 2202 patients (885 (40.2%) men and 1317 (59.8%) women) underwent TSA for pituitary disease during the study period. The mean age was 45 years. Among the 2202 patients, 44 had hyperthyroidism, whereas 218 had hypothyroidism. Among the 44 patients with hyperthyroidism, 9 (20.4%) were confirmed to have Graves’ disease, and 5 (11.4%) were confirmed to have transient thyrotoxicosis. There was no toxic multinodular goiter or toxic adenoma case in this study. Among the 218 patients with hypothyroidism, 53 (24.3%) were confirmed to have primary hypothyroidism. Central thyroid disease was highly common in patients with hyperthyroidism (68.2%) and hypothyroidism (75.7%) (Figure 1).

### 3.2. Prevalence of Thyroid Disease According to Sex

The characteristics of the study patients are shown in Table 1. Upon classification according to sex, the average age was higher in men than in women (*p* < 0.001). The prevalence rates of nonfunctioning pituitary adenomas (NFPAs) and GH-secreting pituitary adenomas were higher in men than in women; contrastingly, the prevalence rates of prolactin (PRL)-secreting pituitary adenomas and adrenocorticotropic hormone (ACTH)-secreting pituitary adenomas were higher in women than in men (all *p* < 0.05, Table 1). 

The prevalence of hyperthyroidism was similar in men and women; however, central hypothyroidism was significantly more common in men than in women. Among the 74 patients with post-thyroidectomy status, 59 had thyroid cancer, and 11 had benign nodules; the reasons for undergoing surgery were unclear (no available data) for four patients. Except for 21 patients who underwent thyroid surgery at other hospitals, for whom no information on histological type was available, almost all patients had papillary thyroid carcinomas (*n* = 35). There were two follicular thyroid carcinoma patients and one double primary (papillary thyroid carcinoma + medullary thyroid carcinoma) carcinoma patient. The most common histological type (94.9%) in Korea is papillary carcinoma (94.9%) [13], and there was no specific thyroid cancer type associated with pituitary tumors. The prevalence of post-thyroidectomy status was significantly higher in women than in men (Table 1).

### 3.3. Prevalence of Thyroid Disease According to Age

Upon classification according to age, hyperthyroidism was more common in younger patients than in older patients. Furthermore, the prevalence of central hyperthyroidism was significantly different between younger and older patients (*p* for trend = 0.039, Table 2). Hypothyroidism was more common in older patients than in younger patients. The prevalence rates of both primary and central hypothyroidism increased significantly with age. The prevalence of post-thyroidectomy status did not differ according to age.

### 3.4. Prevalence of Thyroid Disease According to the Type of Pituitary Disease

The prevalence of thyroid disease according to the type of pituitary disease is shown in Table 3. As expected, central hyperthyroidism was closely related to TSH-secreting tumors. Two patients were found to have TSH-secreting pituitary adenomas after total thyroidectomy for thyroid cancer. These two patients had undergone thyroidectomy 4 months and 12 years before TSA. They showed no clinical manifestations of TSH suppression, despite receiving increased doses of thyroid hormone. TSH-secreting pituitary adenomas were confirmed by further evaluation.

In total, the pituitary pathologic samples from 134 (6.1%) patients showed positive results for TSH. Among these patients, only 26 (19.4%) exhibited the clinical features of central hyperthyroidism. Positive TSH staining was also observed in 1/14 cases of primary hyperthyroidism, 2/53 cases of primary hypothyroidism, 4/165 cases of central hypothyroidism, and 2/74 cases of post-thyroidectomy status. Among the 30 patients with confirmed central hyperthyroidism, 26 showed positive results for TSH. However, the central hyperthyroidism pattern was also observed in four cases with negative TSH staining.

**Figure 2:**
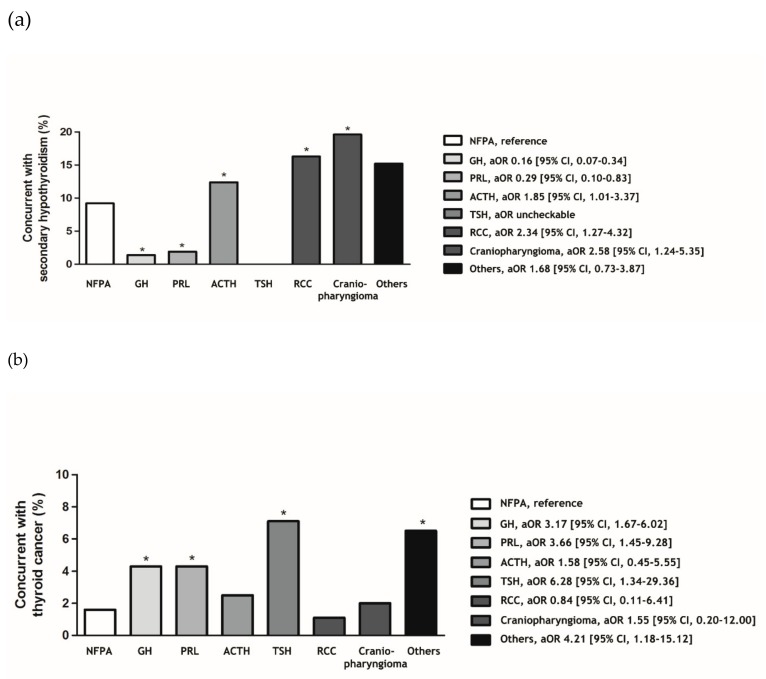
The concurrence rate and odds ratios of central hypothyroidism and thyroid cancer according to type of pituitary disease. Multiple logistic regression analysis was performed to evaluate the concurrent risk of (**a**) central hypothyroidism and (**b**) thyroid cancer. The multiple logistic regression model was adjusted for age and sex. Abbreviations: NFPA: nonfunctioning pituitary adenoma; GH: growth hormone-secreting pituitary adenoma (including GH and PRL co-secreting pituitary adenoma); PRL: prolactin-secreting pituitary adenoma; ACTH: adrenocorticotropic hormone-secreting pituitary adenoma; TSH: thyroid-stimulating hormone-secreting pituitary adenoma (including GH and TSH co-secreting pituitary adenoma); RCC: Rathke’s cleft cyst. * *p*-values < 0.05.

The prevalence of pituitary disease differed according to age and sex. Therefore, its frequency was analyzed after adjusting for age and sex. Central hypothyroidism was more common in patients with ACTH-secreting pituitary adenomas (aOR 1.85, 95% CI 1.01–3.37), Rathke’s cleft cysts (aOR 2.34, 95% CI 1.27–4.32), and craniopharyngiomas (aOR 2.58, 95% CI 1.24–5.35; Figure 2) than in those with NFPAs, after adjusting for age and sex. Contrarily, thyroid cancer had an increased prevalence in patients with GH-secreting pituitary adenomas (aOR 3.17, 95% CI 1.67–6.02), PRL-secreting pituitary adenomas (aOR 3.66, 95% CI 1.45–9.28), and TSH-secreting pituitary adenomas (aOR 6.28, 95% CI 1.34–29.36) (Figure 2).

## 4. Discussion

Thyroid disorders are mainly caused by the thyroid itself but are occasionally caused by pituitary disease. Although the treatment and prognosis of these disorders differ according to their causes, it is difficult to distinguish between the two causes, especially when these disorders occur together. In this study, we found that over 20% of thyroid disorders were primary thyroid disorders that occurred with pituitary disease. In addition, the prevalence of central hypothyroidism was higher in patients with ACTH-secreting pituitary adenomas, Rathke’s cleft cysts, and craniopharyngiomas than in those with NFPAs. Contrastingly, thyroid cancer showed an increased prevalence in patients with GH-, PRL-, and TSH-secreting pituitary adenomas.

In this study, pituitary disease was accompanied by hyperthyroidism, hypothyroidism, and post-thyroidectomy status in 2%, 9.9%, and 3.4% of patients, respectively. The prevalence rates of thyroid-origin thyroid diseases such as primary hyperthyroidism, primary hypothyroidism, and thyroid cancer were 0.7%, 2.5%, and 2.7%, respectively. The prevalence rates of hyperthyroidism and hypothyroidism have varied among various studies. According to the Korea National Health and Nutrition Examination Survey conducted from 2013 to 2015, the prevalence rates of overt hyperthyroidism and hypothyroidism were 0.5% and 0.7%, respectively [14]. For ease of comparison, the prevalence of patients undergoing treatment for hypothyroidism and hyperthyroidism in the Korean general population in 2015 is shown in Table S1 [15] (0.3% for hyperthyroidism and 1.6% for hypothyroidism). Considering that the 5-year prevalence rate of thyroid cancer has not exceeded 1% in the whole Korean population (based on the 2016 survey) [16], the prevalence rates of primary hyperthyroidism, primary hypothyroidism, and thyroid cancer tended to be higher in our study population than in those from previous general population studies. However, when comparing the results of the current and previous studies, we need to consider the large differences in several characteristics of the study population, such as age and gender. Because the national database provides data that is based on results of screening thyroid function tests, the prevalence of thyroid disease may be lower than that of pituitary disease.

Previous general population studies have reported high prevalence rates of hyperthyroidism in women and older patients [14,15,17]. The prevalence of hyperthyroidism was also high in females in this study. However, hyperthyroidism, especially central hyperthyroidism, was more common in younger patients than in older patients in our study population. This might be related to the incidence of TSH-secreting pituitary adenomas that mostly develop in the fifth and sixth decades of life [18]. Previous general population studies showed that the prevalence of hypothyroidism was greater in females and that it increased with age (Table S2) [14,15,17,19]. In our study, the prevalence of hypothyroidism increased with age but was not greater in females. This may be explained by the fact that macroadenomas are more common in men; therefore, men had high chances of developing central hypothyroidism [20,21].

In our study, pathologic data on pituitary disease showed that samples from 134 (6.1%) patients showed positive staining for TSH. However, most patients with positive TSH staining did not show clinical features of central hyperthyroidism. This discrepancy seemed to be related to clinically nonfunctioning or entrapped residual cells. Because pituitary disease often shows inconsistent staining patterns and clinical features, pituitary disease is classified according to clinical function or hormone production [22]. When we examined the differences in prevalence rates of thyroid disease according to the type of pituitary disease, central hypothyroidism was more common in patients with ACTH-secreting pituitary adenomas, Rathke’s cleft cysts, and craniopharyngiomas than in those with NFPAs. In cases of ACTH-secreting pituitary adenomas, excess glucocorticoid levels could have caused the blunted response of TSH [23]. Central hypothyroidism accompanied by Rathke’s cleft cysts [24] or craniopharyngiomas [25] has often been reported. These lesions were thought to be larger than those of other pituitary diseases at the time of surgery because of late detection; however, our obtained data (without data on the size or extent of pituitary disease) were not relevant for pertinent analysis. 

Contrastingly, thyroid cancer had higher prevalence rates in patients with GH-, PRL-, and TSH-secreting pituitary adenomas. An increase in GH levels has been reported to be associated with thyroid cancer and various carcinomas [6]. Acromegaly increases cancer risk, and there is some evidence that GH and IGF-1 are important contributors to thyroid cancer in patients with acromegaly [26]. Moreover, TSH-secreting pituitary adenomas with thyroid cancer have been reported frequently [5]. A possible role of TSH hypersecretion in the development of thyroid tumors was suggested in previous studies [27]. Tam et al. reported that patients with prolactinomas showed increased thyroid nodule frequency and thyroid volume, and increasing evidence is available on the role of prolactin in the development of various cancers [28]; however, the relationship between prolactin levels and cancer risk is still controversial. 

There were several limitations in this study. First, we selected patients who underwent TSA at a tertiary hospital, and this could have resulted in selection bias. However, research on a rare disease is difficult with only general information, such as that provided by the National Health Information database [29]. Second, this study was a cross-sectional study; therefore, a causal relationship could not be assessed. In addition, a direct comparison of the prevalence of thyroid disorders between the study and general populations was not suitable, considering the differences in the method of investigation and the study population. Third, thyroid ultrasonography was routinely performed only in patients with acromegaly. This could have resulted in underestimation of the prevalence of thyroid cancer in patients with other types of pituitary diseases. Furthermore, information on detailed mass size was not clear in the database of this study; thus, we could not analyze the relationship between these parameters and central hypothyroidism. Finally, considering the design and disease definition of this study, it was difficult to deal with the details of subclinical hyperthyroidism and subclinical hypothyroidism that may be missed in medical history taking. Additionally, the possibility of other causes other than thyroid or pituitary origin, including hypothalamic disorders or newly developed thyroid disease following TSA, has been difficult to ascertain due to the limitations of our database. This study was based on data collected up to the time of TSA; therefore, additional research is needed on the risk of thyroid cancer after TSA.

Our study also has various strengths. We enrolled a large population of patients undergoing TSA for pituitary disease over a 10-year period, and we evaluated the prevalence of thyroid disease accompanied by pituitary disease, which is occasionally encountered in the clinical field, especially by endocrine specialists. Furthermore, collection of thyroid function test data and pathologic confirmation were possible because all patients had undergone surgery. In general, prevalence studies are conducted on nationwide population-based cohorts [15]. However, pituitary disease is rare [30], and detailed nationwide information is not available for the accompanying diseases. Our pituitary tumor center is one of the largest and most representative pituitary tumor centers in Korea [17,31,32]. We analyzed medical records and were able to obtain data that were more accurate than those from the National Health Information database. Through this, we could confirm the association of thyroid disease with various types of pituitary diseases. To our knowledge, this is the first study aimed at evaluating the prevalence of thyroid disease in patients who underwent TSA for pituitary disease.

In conclusion, the prevalence of thyroid disease tended to be higher in our study than in previous general population studies, and the prevalence varied according to the type of pituitary disease. We should be aware that pituitary disease sometimes accompanies thyroid disease, and this accompanied thyroid disease may have a different underlying cause than what was originally suspected [33]. Pituitary diseases may mask thyroid disorders, and focusing on the pituitary problem can neglect the thyroid disease.

## Figures and Tables

**Table 1:** Characteristics of the study population and prevalence of thyroid disease.

	Total	Male	Female	*p* Value
	(*n* = 2202)	(*n* = 885)	(*n* = 1317)	
Age (year)	45.0 ± 13.5	47.4 ± 13.7	44.3 ± 13.2	<0.001
Type of pituitary tumor				
NFPA	1167 (53.0)	521 (58.9)	646 (49.1)	<0.001
GH	488 (22.2)	223 (25.2)	265 (20.1)	0.005
PRL	209 (9.5)	19 (2.1)	190 (14.4)	<0.001
ACTH	121 (5.5)	22 (2.5)	99 (7.5)	<0.001
TSH	28 (1.3)	12 (1.4)	16 (1.2)	0.772
RCC	92 (4.2)	37 (4.2)	55 (4.2)	0.996
Craniopharyngioma	51 (2.3)	27 (3.1)	24 (1.8)	0.060
^a^Others	46 (2.1)	24 (2.7)	22 (1.7)	0.094
Hyperthyroidism, n (%)	44 (2.0)	14 (1.6)	30 (2.3)	0.253
Primary, n (%)	14 (0.7)	2 (0.2)	12 (1.0)	0.056
Central, n (%)	30 (1.4)	12 (1.4)	18 (1.4)	0.904
Hypothyroidism, n (%)	218 (9.9)	102 (11.5)	116 (8.8)	0.036
Primary, n (%)	53 (2.5)	16 (1.8)	37 (3.0)	0.103
Central, n (%)	165 (7.8)	86 (9.8)	79 (6.3)	0.003
Post-thyroidectomy status, n (%)	74 (3.4)	11 (1.2)	63 (4.8)	<0.001
Cancer, n (%)	59 (2.7)	10 (1.1)	49 (3.7)	<0.001
Benign nodule, n (%)	11 (0.5)	0 (0.0)	11 (0.8)	0.004
Unknown reason, n (%)	4 (0.2)	1 (0.1)	3 (0.2)	>0.999

Data are expressed as means ± standard deviations for normally distributed continuous variables and as numbers (%) for categorical variables. Abbreviations: NFPA: nonfunctioning pituitary adenoma; GH: growth hormone-secreting pituitary adenoma (including GH and PRL co-secreting pituitary adenoma); PRL: prolactin-secreting pituitary adenoma; ACTH: adrenocorticotropic hormone-secreting pituitary adenoma; TSH: thyroid-stimulating hormone-secreting pituitary adenoma (including GH and TSH co-secreting pituitary adenoma); RCC: Rathke’s cleft cyst. ^a^Others: Chordoma, tumors metastatic to the pituitary gland, lymphocytic hypophysitis, meningioma, germinoma, etc.

**Table 2:** Prevalence of thyroid disease according to age.

	Total	~39	40~59	60~	*p* for Trend
	(*n* = 2202)	(*n* = 802)	(*n* = 1058)	(*n* = 342)	
Hyperthyroidism, n (%)	44 (2.0)	21 (2.6)	22 (2.1)	1 (0.3)	0.017
Primary, n (%)	14 (0.6)	6 (0.7)	8 (0.8)	0	0.231
Central, n (%)	30 (1.4)	15 (1.9)	14 (1.3)	1 (0.3)	0.039
Hypothyroidism, n (%)	218 (9.9)	54 (6.7)	114 (10.8)	50 (14.5)	<0.001
Primary, n (%)	53 (2.4)	12 (1.5)	26 (2.5)	15 (4.4)	0.005
Central, n (%)	165 (7.5)	42 (5.2)	88 (8.3)	35 (10.2)	0.001
Post-thyroidectomy status, n (%)	74 (3.4)	19 (2.4)	42 (4.0)	13 (3.8)	0.108
Cancer, n (%)	59 (2.7)	16 (2.0)	32 (3.0)	11 (3.2)	0.165
Benign nodule, n (%)	11 (0.5)	2 (0.2)	8 (0.8)	1 (0.3)	0.573
Unknown reason, n (%)	4 (0.2)	1 (0.1)	2 (0.2)	1 (0.3)	0.547

Data are expressed as numbers (%) for categorical variables.

**Table 3:** Prevalence of thyroid disease according to the type of pituitary disease.

	Total	NFPA	GH	PRL	ACTH	TSH	RCC	Craniopharyngioma	Others ^a^	*p*-Value
	(*n* = 2202)	(*n* = 1167)	(*n* = 488)	(*n* = 209)	(*n* = 121)	(*n* = 28)	(*n* = 92)	(*n* = 51)	(*n* = 46)	
Hyperthyroidism, n (%)	44 (2.0)	12 (1.0)	5 (1.0)	1 (0.5)	0	26 (92.9)	0	0	0	<0.001
Primary, n (%)	14 (0.7)	9 (0.8)	5 (1.0)	0	0	0	0	0	0	0.660
Confirmed as Graves’ disease	9 (0.4)	8 (0.7)	1 (0.2)							
Confirmed as transient thyrotoxicosis	5 (0.2)	1 (0.1)	4 (0.8)							
Central, n (%)	30 (1.4)	3 (0.3)	0	1 (0.5)	0	26 (92.9)	0	0	0	<0.001
Hypothyroidism, n (%)	218 (9.9)	147 (12.6)	9 (1.8)	7 (3.3)	18 (14.9)	0	16 (17.4)	11 (21.6)	10 (21.7)	<0.001
Primary, n (%)	53 (2.5)	40 (3.4)	2 (0.4)	3 (1.4)	3 (2.5)	0	1 (1.1)	1 (2.0)	3 (6.5)	0.008
Central, n (%)	165 (7.8)	107 (9.2)	7 (1.4)	4 (1.9)	15 (12.4)	0	15 (16.3)	10 (19.6)	7 (15.2)	<0.001
Post-thyroidectomy status, n (%)	74 (3.4)	26 (2.2)	27 (5.5)	10 (4.8)	4 (3.3)	2 (7.1)	1 (1.1)	1 (2.0)	3 (6.5)	0.014
Cancer, n (%)	59 (2.7)	19 (1.6)	21 (4.3)	9 (4.3)	3 (2.5)	2 (7.1)	1 (1.1)	1 (2.0)	3 (6.5)	0.012
Benign nodule, n (%)	11 (0.5)	5 (0.4)	5 (1.0)	0	1 (0.8)	0	0	0	0	0.633
Unknown reason, n (%)	4 (0.2)	2 (0.2)	1 (0.2)	1 (0.5)	0	0	0	0	0	0.977

Data are expressed as numbers (%) for categorical variables Abbreviations: NFPA: nonfunctioning pituitary adenoma; GH: growth hormone-secreting pituitary adenoma (including GH and PRL co-secreting pituitary adenoma); PRL: prolactin-secreting pituitary adenoma; ACTH: adrenocorticotropic hormone-secreting pituitary adenoma; TSH: thyroid-stimulating hormone-secreting pituitary adenoma (including GH and TSH co-secreting pituitary adenoma); RCC: Rathke’s cleft cyst ^a^ Chordoma, tumors metastatic to the pituitary gland, lymphocytic hypophysitis, meningioma, germinoma, etc.

## Supplementary Materials

The following are available online at https://www.mdpi.com/2077-0383/8/8/1142/s1, Table S1: Clinical characteristics of the study population according to the type of pituitary disease, Table S2: Prevalence of patients undergoing treatment for hypothyroidism and hyperthyroidism in the general Korean population in 2015 (Adapted and modified from Kwon et al. Endocrinol Metab 2018; 33:260-267).
