# Supplementary material for: Prevalence of Thyroid Disease in Patients Surgically Treated for Pituitary Disease
Source: J Clin Med. 2019 Jul 31;8(8):1142. doi: 10.3390/jcm8081142 (PMC6722506; doi:10.3390/jcm8081142)
Supplement: Supplementary file 1 [file jcm-08-01142-s001.pdf]

Table S1. Clinical characteristics of the study population according to the type of pituitary disease.

	Total (n = 2202)	NFPA (n = 1167)	GH (n = 488)	PRL (n = 209)	ACTH (n = 121)	TSH (n = 28)	RCC (n = 92)	Cranio-pharyngioma (n = 51)	Others[a] (n = 46)	p-value
Age	45.0 ± 13.5	49.7 ± 12.7	44.0 ± 11.7	32.6 ± 8.4	39.7 ± 13.4	41.7 ± 8.5	38.0 ± 14.2	43.0 ± 14.4	51.6 ± 15.4	<0.001
Female, n (%)	1317 (59.8)	646 (55.4)	265 (54.3)	190 (90.9)	99 (81.8)	16 (57.1)	55 (59.8)	24 (47.1)	22 (47.8)	<0.001
Microadenoma, n (%)	373 (16.9)	50 (4.3)	119 (24.4)	121 (57.9)	79 (65.3)	4 (14.3)				<0.001
Macroadenoma, n (%)	1640 (74.5)	1117 (95.7)	369 (75.6)	88 (42.1)	42 (34.7)	24 (85.7)				
Others, n (%)	189 (8.6)						92 (100.0)	51 (100.0)	46 (100.0)	

Data are expressed as means ± standard deviations for normally distributed continuous variables and as numbers (%) for categorical variables Abbreviations: NFPA: nonfunctioning pituitary adenoma; GH: growth hormone-secreting pituitary adenoma (including GH and PRL co-secreting pituitary adenoma); PRL: prolactin-secreting pituitary adenoma; ACTH: adrenocorticotropic hormone-secreting pituitary adenoma; TSH: thyroid-stimulating hormone-secreting pituitary adenoma (including GH and TSH co-secreting pituitary adenoma); RCC: Rathke's cleft cyst [a]Chordoma, tumors metastatic to the pituitary gland, lymphocytic hypophysitis, meningioma, germinoma, etc.

Table S2. Prevalence of patients undergoing treatment for hypothyroidism and hyperthyroidism in the general Korean population in 2015 (Adapted and modified from Kwon et al. Endocrinol Metab 2018; 33:260-267).

	Hyperthyroidism			Hypothyroidism		
	**Total**	**Male**	**Female**	**Total**	**Male**	**Female**
Total	0.3	0.2	0.4	1.6	0.5	2.7
~39	0.2	0.1	0.2	0.5	0.2	0.9
40~59	0.4	0.3	0.5	2.3	0.7	4.0
60~	0.4	0.2	0.5	3.1	1.3	4.6

Values are expressed as percentages.
